# 非小细胞肺癌手术入路及切除方式研究进展

**DOI:** 10.3779/j.issn.1009-3419.2018.09.08

**Published:** 2018-09-20

**Authors:** 焕松 杨, 巨伟 牟

**Affiliations:** 100000 北京，中国医学科学院肿瘤医院胸外科 Department of Thoracic Surgery, Cancer Hospital of Chinese Academy of Medical Science, Beijing 100000, China

**Keywords:** 肺肿瘤, 电视辅助胸腔镜手术, 手术入路, 手术方式, 并发症, Lung neoplasms, Vidio-assisted thoracic surgery, Surgical approaches, Operation method, Complication

## Abstract

随着疾病谱的改变，肺癌的发病率和死亡率在全球范围内一直居高不下，自从外科干预被应用于肺癌的治疗，其地位日益提高，目前以外科手术为主的综合治疗已成为肺癌治疗的首选方案，外科手术入路和术式种类繁多，并且新的技术不断出现，本文拟总结不同手术方式和手术入路的研究进展。随着手术软硬件技术的发展和微创理念深入人心，胸腔镜微创手术较传统开胸手术为肺癌患者带来了更多的福音，手术方式的改变也可更大限度保留肺组织，提高患者的生存质量，相信随着各类手术适应证和手术方式的进一步规范，微创胸腔镜手术会给肺癌患者带来更多的益处。

## Abstract (en)

With the change of the spectrum of disease, the incidence and mortality of non-small cell lung cancer (NSCLC) has been high in global scale, since surgical intervention was applied to treat lung cancer, its status is increasing day by day, at present comprehensive treatment leaded by surgery has become the preferred scheme for NSCLC, there are many different kinds of surgical approaches and operation methods of disease, and the new technologies appear constantly, the paper aim to summarize the research progress of different operationmethods and surgical approach. With the development of software and hardware technology and the concept of minimally invasive thoracoscopic surgery was received by more people, minimally invasive thoracoscopic surgery has brought more benifit than traditional thoracotomy for lung cancer patients, minimally invasive thoracoscopic surgery reserve more lung tissue and improve the survival quality of patients due to better pulmonary function, we believe that minimally invasive thoracoscopic surgery can bring more benefits to people with surgical indications and surgical procedure further standardization.

世界范围内肺癌的发病率和死亡率均居男性恶性肿瘤的第一位，女性发病率居第三位，死亡率则居第二位^[1]^。在中国，肺癌发病率和死亡率均列恶性肿瘤之首，分别为48.32/10万和39.27/10万。肺癌已经是危害人类健康的头号杀手，而且呈继续上升趋势^[2]^。因此，对于肺癌的治疗一直都是医疗工作者的研究重点。

目前，治疗肺癌的主要临床方法包括外科手术切除病灶、化疗、放疗、生物工程药物靶向治疗配合的多学科综合治疗，其中外科手术目前仍是治疗非小细胞肺癌（non-small cell lung cancer, NSCLC）的重要方法^[3]^。外科手术治疗肺部肿瘤最早可追溯到1933年^[4]^，经过多年的发展，手术方式已经包括开胸手术、多孔及单孔胸腔镜辅助手术、新兴的达芬奇机器人手术和剑突下胸腔镜手术等多种形式^[4]^，各种手术方式具有不同的优缺点及应用条件。

## 1 肺癌外科手术入路

### 1.1 传统开胸手术的介绍

早期的肺癌切除手术经历了前外侧切口、外侧切口及后外侧切口的发展过程，配合横断肋骨甚至胸骨正中切开等来满足特殊部位切除的需要，患者并发症严重，并发症发生率高，术后疼痛严重及肩关节运动功能受损严重，严重影响生活质量，20世纪80年代出现了非离断肌肉的开胸手术，对于后外侧切口患者只离断背阔肌的1/3，对于肋间切口患者基本不离断肌肉，新型Navarro牵开器的出现，只需4英寸的切口和2英寸的肋间牵开距离，使得原有圆柱形视野扩展为圆锥形视野^[5]^，既保证了手术的顺利进行，又减轻了术后疼痛，研究表明该手术可完成绝大部分临床评估可行外科干预患者的手术，并且与传统的大切口手术相比无明显生存差异^[1]^。如今我们提到的开放手术已经不是传统意义上的后外侧大切口甚至切除肋骨，最小限度地分离肋间肌肉和肋骨配合运用先进的操作设备已成为如今开放手术的常态^[6]^。开胸手术的优势在于术者可以直接触及肺组织，这在肿瘤组织侵犯血管和新辅助化疗后解剖平面粘连消失等患者的手术中尤为重要。

### 1.2 电视辅助胸腔镜手术（video-assisted thoracic surgery, VATS）手术的发展现状

VATS在1992年被首次应用于治疗肺癌^[7]^，自此以后，越来越多的外科医生试图采取这一技术以期减少手术损伤，目前对于国内胸腔镜的使用量并没有详细的统计数据，不过据估计，在2016年中国有一半的肺叶切除手术是通过胸腔镜完成的^[3]^。胸腔镜手术已经不再是一个新鲜事物或一种趋势，而是已经成熟应用于临床多种胸部疾病治疗。欧洲胸外科协会的一项纳入287, 771例患者的研究表明胸腔镜组在心肺并发症、肺不张、通气功能障碍和切口感染等主要术后并发症方面的发生率明显低于开放手术组，术后疼痛和术后住院时间均优于开放手术组，还特别提出在70岁以上人群、营养不良患者及伴有心功能不全患者中，胸腔镜组术后并发症和术后住院时间均优于开放手术组^[8]^，这也为身体条件不能满足传统手术要求的患者提供了手术机会和延长生存期的可能。甚至有双盲随机试验研究表明，VATS对于治疗早期NSCLC是最佳选择，因为它能在术后第1年内减少患者疼痛感，从而提高患者生活质量^[9]^，原因可能在于随着切口的减小，炎症反应随之降低^[10]^，于是有人提出，是否随着切口的越来越小获益会增加，于是在三孔VATS的基础上单操作孔和单孔VATS应运而生。单孔VATS于2004年被Rocco首次用于肺楔形切除，于2011年在第一次被刚萨雷斯报道应用于临床肺叶切除术和淋巴结清扫^[11]^，近几年得到了迅速发展，已经应用于气胸、感染性疾病、良恶性肿瘤等疾病的治疗。多项研究^[12, 13]^表明在术后并发症、手术时间、淋巴结清扫、胸管引流时间、住院时间和失血量等方面单孔VATS优于多孔VATS。有研究^[14]^分析了8篇文章，仅其中1篇认为传统胸腔镜优于单孔，其余文章表明单孔优于多孔。因此，总的研究结果显示，单孔胸腔镜在治疗NSCLC方面并不逊色于传统VATS手术，甚至在某些方面优于传统胸腔镜手术。但是，目前单孔VATS仍然存在一些不足，第一，单孔VATS要求所有操作在一个孔内进行，所以普通胸腔镜器械会互相干扰，而专业单孔器械价格比较昂贵，会加重患者经济负担，另外部分本身肋间隙狭窄患者也会增加手术难度，甚至造成肋间神经损伤而出现术后疼痛。第二，对于肿瘤侵及胸壁、大血管或神经或者肿瘤坏死易出血等肿瘤情况复杂的患者，不应考虑单孔^[15]^。第三，单孔胸腔镜的开展情况在世界各地情况并不一致，在中国、西班牙、日本等国开展较多而在英国等欧洲国家开展较少^[14]^，所以全世界范围内推广单孔VATS技术还面临一定挑战。第四，单孔VATS手术缺乏详尽的应用共识和操作规范，少数人盲目追求单孔手术，不利于患者的安全和疾病的治疗。第五，各项研究结果中，手术时间彼此差距较大，这可能与术者的熟练度有关，因此提高术者水平、确定统一的手术程序和标准对于提高和发展这项技术至关重要^[16]^。尽管单孔胸腔镜肺叶切除技术刚刚兴起数年，仍需大量的数据和时间来证明其较多孔胸腔镜手术的优越性，但其安全性及可行性已得到证实，并且在气管、血管、隆突重建和袖式切除等复杂手术中也已得到应用^[17]^。

### 1.3 剑突下VATS的优势

单孔胸腔镜手术近几年快速发展，但由于操作时肋间神经的破坏和卡压导致术后患者肋间神经疼痛和胸壁麻木感一直是一个未能解决的棘手问题^[18]^，不同文献显示关于胸腔镜手术后慢性疼痛的发生率在11%-80%之间不等^[19]^，而经剑突下进行胸腺切除时，由于不经过肋间隙则不会出现术后肋间慢性疼痛的问题^[20]^，于是在2014年第一次有人尝试通过剑突下4 cm长切口进行肺叶切除术^[21]^，之后有多项研究表明剑突下腔镜术后慢性疼痛较经肋间手术明显减轻^[18]^，这一手术方式虽然成熟应用于胸腺切除，应用于肺部手术仍处于探索阶段，仍然需要更多的中心和更好的随机对照实验来验证其短期并发症和远期生存率与传统VATS手术是否存在差异。

### 1.4 机器人手术的现状

为了解决胸腔镜手术学习曲线不稳定、手眼协调性差、设备灵活性差、二维视图下人体解剖形态失真等问题，机器人手术系统应运而生^[7, 22]^，达芬奇机器人手术系统是目前唯一一个被广泛应用于各类外科手术的完整机器人手术系统，经过几十年的发展，达芬奇机器人系统已经发展到第四代（standand、s、si、x），每一代都在原有基础上对安全性、灵活性和功能方面进行了提升^[23]^，达芬奇机器人手术系统由控制台、床旁机械臂及成像系统组成，外科医生在无菌区以外通过控制台操纵无菌区内的机械臂，成像系统则集中了机器人系统的核心处理器和成像系统，通过高度敏感的传感器，医生6 HZ大小以内的震动可被有效滤除保证手术的准确性，机械臂上的高分辨率三D摄像头对手术视野放大10倍-15倍，从而提供更加清晰的手术画面，保证微细结构的安全处理^[7, 23]^，机器人手术入路的选择在不同机构中，甚至同一机构不同时期也不尽相同，Gharagozloo等^[24]^描述了一种混合技术，3个机器臂被安置在第5、第6和第8肋间隙用于分离肺门组织，然后外科医生返回手术台继续离断血管气管和肺组织，Jang等^[25]^把镜头放置在腋中线第七肋间，一个机械臂放置在腋后线第6或7肋间隙，另一个放置在第5肋间隙乳房下缘，Cerfolio等^[26]^则把4个机械臂全部放置在第七肋间隙腋中线与脊柱旁线之间，对此目前尚无统一的标准，也没有研究数据显示机械臂不同入路对于手术效果的影响。目前机器人手术系统病人的选择主要为Ⅰ期和Ⅱ期NSCLC患者^[24, 26]^，Veronesi等^[27]^还提出肿瘤最大径要小于5 cm的患者才适合机器人手术。那么机器人手术与开胸手术及VATS手术究竟有何异同呐？2011年的一项回顾性对比研究比较了106例机器人肺叶切除术患者和318例传统开胸肺叶切除术患者，结果表明患者的术中和术后并发症和死亡率并没有明显差别，而机器人组患者住院时间减少，患者主观生活质量明显提高，但是机器人手术组手术时间较开放组明显延长（2.2 h *vs* 1.5 h）^[28, 29]^；同年的另一项研究则比较了40例机器人手术患者、40例初次VATS手术患者和40例既往VATS手术患者，结果显示初次胸腔镜组手术时间与机器人组并无统计学差异，机器人组的中转开胸率明显低于另两组患者，三组之间术后并发症并无明显差异^[25]^。而Marcello和Ricciardi^[7, 23]^的研究表明机器人手术组的手术时间明显长于胸腔镜和开放手术组，但学习曲线表明其要获得可靠的手术技能所需的病例数较传统胸腔镜手术少，所需总手术时间较胸腔镜手术短。手术中淋巴结的清扫数量也是评价手术效果的一项指标，Cerfolio比较了机器人手术和开放手术的淋巴结清扫数目和站数，结果发现淋巴结清扫数目（17 *vs* 15）和站数（3 *vs* 3 N1站，5 *vs* 5 N2站）均无统计学差异^[28]^。Jang等^[25]^对比了机器人手术组、腹腔镜组和既往腹腔镜手术组，发现三者无论是淋巴结清扫数量还是清扫站数均无统计学差异。甚至有研究表明在狭小的操作空间里，机器人系统对于更大范围清扫N1淋巴结和更精确清扫N2淋巴结更有优势^[23]^。作为新兴的手术技术，机器人手术系统也有一些固有的优势，第一，机器人手术是在胸腔完全封闭的情况下进行的，通过注入温暖的二氧化碳气体使胸廓膨胀，这不仅提供充足的操作空间，也避免了柔软的肺组织直接与手术室干燥寒冷的空气接触，并且由于膈肌被气体挤压下移，减少了膈肌误损伤的可能^[30]^。第二，机械臂定义的7个自由度可以完美模拟手部活动，并且应用零度相机，较胸腔镜系统普遍使用的30度相机能更真实地展现解剖结构，从而避免副损伤^[30]^。当然机器人手术高昂的费用问题是不容忽视的，多项研究表明机器人手术费用明显高于胸腔镜手术^[31]^，其次外科医生通过控制台操作器械，而即使第四代机器人也无法提供完全一致的力反馈，这对一个外科医生来说又是影响手术安全性的重要因素^[7]^，最后，目前仍缺乏大型随机对照实验验证其远期生存情况和生活质量与传统VATS手术的差异。

## 2 肺癌手术切除方式的改变

在1995年，肺叶切除及纵隔淋巴结清扫被定义为早期NSCLC手术治疗的金标准^[32]^，随着外科手术微创理念的发展，外科医生对于手术切除范围也有了新的探索，对于早期肺癌的亚肺叶切除的探索悄然兴起，亚肺叶切除包括楔形切除和肺段切除两种形式，前者根据病变所在位置决定手术切除范围，并不离断血管和气管，也不进行淋巴结清扫，而后者则依据肿瘤所在位置切除相应肺段，离断血管和气管并进行淋巴结清扫^[33, 34]^。目前VATS亚肺叶切除主要应用于：①Ⅰa期周围型NSCLC：直径≤2 cm，位于肺外1/3，局限于单一肺段内，切缘距离/肿瘤直径比值> 1；N1组、N2组淋巴结术中冰冻病理阴性；②肺内磨玻璃影（ground-glass opacity, GGO）：直径 < 1.5 cm的纯GGO可行楔形切除，如GGO成分不足50%，或距肺边缘 > 2 cm者，应行肺段切除；③合并不同肺叶内小结节需同期手术切除；④心肺功能较差，FEV_1_% < 50%患者；⑤肺叶切除术后再发癌患者。一项随机对照实验^[35]^对比了肺叶切除与肺段及楔形切除的远期差异，实验的目的是为了证明肺段及楔形切除的可行性，结果却出人意料，结果表明肺段和楔形切除组的局部复发率是肺叶切除组的3倍，总体生存期也较肺叶组下降。Cao等^[36]^分析了56篇文章，他根据手术方式将研究分为三个组，亚肺段组对比肺叶组、肺段组对比肺叶组以及楔形组对比肺叶组，同时每组又分为主动选择组、被动选择组和未区分组三个亚组，研究的评价指标为总生存时间。在主动选择组中，亚肺叶组与肺叶组并无明显区别，而在被动选择组和未区分组中肺叶切除组的生存时间明显优于亚肺叶切除组；在主动选择组和未区分组，肺段和肺叶切除组生存期并无明显差别，而被动选择组，肺段切除组的生存期明显劣于肺叶切除组；主动选择组的楔形切除与肺叶切除也并无明显差异，被动选择组和未区分组的生存期这明显短于肺叶切除组。由此可见影响患者生存期的主要因素并非切除方式本身，如何有效的选择合适的患者接受合适的切除方式至关重要。也有研究认为亚肺叶切除手术应该保证切缘距肿瘤边缘距离应 > 2 cm或大于肿瘤最大直径，才能保证手术的效果^[32]^，也有学者提出由于直径 < 2 cm的肿瘤N1和N2淋巴结阳性率分别为5.3%和6.6%，所以对于亚肺叶切除也应常规清扫淋巴结，但是同时，由于楔形切除并没有统一的解剖界限，所以如何划定淋巴结清扫区域又是一项新的挑战。

## 3 总结

随着科技的进步发展，各种各样先进手术设备的发明和微创理念的深入人心，手术操作者越来越关注先进技术和新手段在临床中的应用，胸腔镜手术由于其较开胸手术创伤小、术后恢复快、并发症少、远期效果较传统手术无明显差异等优点，得到了临床和患者的认可从而快速发展，替代了部分传统开放手术，在此基础上的单孔胸腔镜、剑突下入路胸腔镜近几年也因创伤小、恢复快等优势而受到广泛关注，关于手术切除方式也不断有人提出新的理念，以求有效切除病变的同时尽可能的保留患者的肺功能，肺段切除和楔形切除被应用于早期NSCLC，并且有研究表明在仔细筛选的人群中行肺段和楔形切除手术，效果与标准的肺叶切除并无明显差异，所以肺段和楔形切除在认真筛选的人群中可以有效治疗疾病、减少并发症并提高生活质量。机器人辅助胸腔镜系统更是在保证手术效果的同时提高了微细结构处理能力和操作精准度，减少了误损伤的可能。在新技术和新方案给患者带来益处的同时也有一些问题亟待解决。第一，由于胸腔镜手术的治疗效果对于手术操作者依赖性大，不同地区和不同中心开展情况有较大差距，因此规范化的培训对于推广胸腔镜并保证手术质量至关重要。第二，尽管胸腔镜手术有着技术本身的优势，但仍然有其局限性，尤其是在病变较晚、胸腔广泛粘连和大出血需要止血的患者中应用起来手术难度较大，因此制定专家共识选择适应证来保证手术安全有效至关重要。第三，剑突下入路胸腔镜手术和机器人手术技术仍处于探索阶段，尽管在少数中心进行了尝试，距离大规模推广仍有很长的路要走，需要更多的前瞻性实验来验证其安全性和有效性。第四，楔形和肺段切除技术虽然在筛选人群中效果与标准肺叶切除无差别，但针对远期生存率和复发率情况并无前瞻性数据支持，而在淋巴结清扫范围方面也缺少官方的指南，只有这些问题得到解决才能使得新技术和新方案为患者带来最大的获益。
